# Factors influencing the progression of post-mortem changes between scene and autopsy

**DOI:** 10.1038/s41598-026-35786-x

**Published:** 2026-01-14

**Authors:** Nina Lanzinger, Marcel A. Verhoff, Christoph G. Birngruber, Lena Lutz

**Affiliations:** Institute of Legal Medicine, Goethe-University Frankfurt, University Hospital, Kennedyallee 104, D-60596 Frankfurt am Main, Germany

**Keywords:** Post-Mortem interval (PMI), Decomposition, Total body score (TBS), Total decomposition score (TDS), Cooling period, Anatomy, Medical research

## Abstract

Temperature is a pivotal factor influencing the progression of the decomposition process of a human cadaver and thus its post-mortem changes. In cases of advanced decomposition, various problems arise in routine forensic work, such as difficulties in estimating the time or cause of death. To understand and evaluate the impact of temperature and other related factors on the progression of post-mortem changes between the post-mortem examination and autopsy, 135 dead bodies with different post-mortem intervals (PMI) and varying storage times between discovery and autopsy were examined. The Total Decomposition Score (TDS) and the Total Body Score (TBS) were used to assess and compare the post-mortem changes, while continuous temperature measurements were taken inside the body bags throughout the cooling phase. It was found that the most important factors leading to an increased progression of the post-mortem changes were a high initial body temperature, few post-mortem changes (low TDS/TBS scores) present at the beginning of the cooling period, insect infestation of a body, and a prolonged duration of the storage period. The establishment of uniform standards for the cooling of bodies in Germany, as well as the recognition and appropriate treatment of cases with an increased risk of rapid progression of the post-mortem changes during the cooling period, has the potential to improve the preservation of forensic evidence.

## Introduction

The post-mortem interval (PMI) refers to the elapsed time between an individual’s death and the subsequent discovery of the body. An extended PMI is closely linked to the progression and extent of post-mortem changes, which can complicate forensic examinations and the determination of the time of death^1,2^. Enhancing our understanding of the progress of decomposition and its driving factors has become increasingly significant due to demographic developments and climate change. Research indicates that demographic and socio-economic factors significantly influence the time between the death of a person and the discovery of their body, i.e., the length of the PMI. Advanced age, along with other factors like reduced mobility, living alone without the support of care institutions or family members, and social disadvantages—like limited financial resources or being part of a minority—can lead to social isolation^3,4^. This, in turn, can result in a delayed discovery of a deceased person. With the population over the age of 80 expected to double in the coming decades^5^, cases of social isolation will also increase. This suggests that an increase in the number of bodies discovered later in time and exhibiting advanced post-mortem changes can be expected. Furthermore, fluctuations in temperature and humidity have been identified as factors that greatly influence the rate of decomposition. It is anticipated that rising global temperatures and increasing humidity levels—as consequences of climate change—will result in a substantial decrease in the time required for complete skeletisation^6^.

Determining the PMI is of considerable significance in each death investigation as it can facilitate a more comprehensive understanding of the circumstances and course of events of a crime^7^. The cooperation between various specialist areas and disciplines in the field of forensic science is most effective in estimating the PMI, determining the circumstances of death, or identifying a body. Police officers collect circumstantial evidence, while forensic pathologists can assess the time and the cause of death. Furthermore, forensic odontologists can compare the ante-mortem and post-mortem dental charts^8^, helping with the identification of bodies in advanced stages of decomposition. Forensic entomologists can estimate a minimum PMI, i.e., the time since the first insect colonization of a body. Moreover, analyses of insect traces can give additional information, including details on the season at the time of death, the post-mortem transfer of a body, and a possible intoxication prior to death^9^. The combination of all these different disciplines is essential for an accurate depiction of the circumstances surrounding a death.

Over the years, a range of methods has been proposed to estimate the PMI. These include, e.g., the study of microbial neoformations^10^ and skeletal muscle proteins^10,11^, the use of collaborative forensic databases^12^, the employment of Bayesian networks^13^, and temperature-based PMI estimations^14^. Further approaches involve the analysis of microbiomes, physiochemical and thanato-chemical markers^15,16^, a more detailed understanding of insect physiology and ecology^9^, the analysis of protein degeneration or the decay of radioisotopes^11,17–19^, the launching of genetic markers^20,21^, and the analysis of bone structures and components^18^. However, despite these efforts, none of the methods has yet yielded sufficient results for calculating the PMI with the level of precision required for legal admissibility or their applicability is limited to specific timeframes or circumstances. Consequently, none of these approaches has yet been universally accepted and implemented on a global scale within the context of forensic science. The quantification of decomposition stages is another important tool to estimate the PMI^19,22^, especially when early post-mortem changes, such as rigor mortis, livor mortis, and algor mortis, can no longer be assessed due to advanced decay. The decomposition process of humans was studied in various environments, considering the local climatic conditions^23–31^. Many attempts have been made to implement a standard or formula to determine the PMI^1,2,12,13,32^ considering different variables influencing the decomposition rate like humidity, ambient temperature, body temperature, body weight, location, season, and pre-existing medical conditions^2,26,27,29,33–38^. Additionally, the role of insects and scavengers in decomposition has been the subject of numerous studies^37,39,40^. However, the formulas are often limited in validity to the specific conditions in which they were developed and tested^17^.

Rather than being shaped by single environmental parameters, decomposition is fundamentally influenced by the dynamic interactions of many variables^25,26,30,33,35,41–43^, e.g., humidity, temperature, soil conditions, shadow, insects, moisture, partial pressure, or acidity. The decomposition process can be divided into two phases: The process of autolysis, catalysed by enzymes, and putrefaction, mediated by bacteria and fungi that originate either from the body’s own microbiome or the external environment^10,39,42,44,45^. These phases vary considerably in terms of sequence and speed^17^; in some instances, the process may attain a plateau phase prior to further progression^22,25,39,46^. PH value, temperature, moisture, and partial pressure of oxygen are four key variables that determine the progression of post-mortem changes^24,32,41,42^. Among these, temperature is recognized as a pivotal factor in the process of decay, although the exact proportion of this component has not yet been conclusively clarified. Megyesi et al.^1^ estimated the influence of temperature and elapsed time on the decomposition process at 80%, while others arrive at controversial results between 25% and 94%^28,30,46,47^.

The objective of quantifying post-mortem changes with a comparable score remains a challenge in the field of forensic medicine since decomposition is a highly variable process influenced by numerous factors that differ globally. To capture decay, a system must encompass as many of these variations as possible while remaining comparable and easy to use. There are numerous approaches to this^1,43,48^, one of the best-known being the Total Body Score (TBS), which was developed by Megyesi et al.^1^. The TBS is used to quantify the extent of decomposition by assigning scores to various body regions based on the progression of post-mortem changes. The sum of these regional scores yields the TBS, providing a standardised measure of overall decomposition. A notable deficiency in the system devised by Megyesi et al.^1^ is that it is rather rigid, viewing decomposition as a linear process, not allowing many variations in the temporal course of decomposition. However, it is user-friendly and has a high inter-observer reliability^49^. Additionally, it has been tested for its usage on digital images^50^. Gelderman et al.^48^ further developed a system based on the aforementioned system from Megyesi et al.^1^. Simultaneously, a separate score is recorded for the different anatomical regions. The Total Decomposition score (TDS) is then calculated by adding these values together. For each body region, there are points from 1 to 6, and each point value is assigned a series of visible post-mortem changes, one or more of which may be present. This facilitates a more flexible evaluation of bodies, especially in cases where multiple characteristics are present concurrently.

Once a body has been found, the mandatory initial post-mortem examination is conducted before the body is placed in cold storage. In cases where the manner of death is considered unnatural or unclear, the intervention of law enforcement authorities becomes necessary. The subsequent course of action is then determined by the public prosecuting office. Meanwhile, the bodies are removed by the mortician and stored in appropriate cooling facilities. In a multitude of cases, the bodies are thereafter transferred to an Institute of Legal Medicine, following the order of an autopsy. There, the bodies are stored in cooling cells until their autopsy is performed. This sequence of proceedings can result in extended periods of cooling. In a study conducted between 2018 and 2020 at the Institute of Legal Medicine in Frankfurt am Main, the period between discovery and autopsy was on average 6.2 days^51^. Another study by Ceciliason et al.^52^ in Sweden reported an average duration of this period of 4 days. During this time, the decomposition can progress^53^, resulting in a loss of forensic evidence, e.g., superficial wounds or bruises. This, in turn, can complicate the determination of the cause of death^4^.

The combined consideration of continuous temperature measurements^51^ alongside other factors that influence post-mortem changes during the period between the discovery of a body and autopsy has not yet been investigated in a sufficient manner. To address this gap, a set comprising a questionnaire and data loggers was implemented. These were to be taken by the attending physicians to the post-mortem examination. On site, the data logger was attached to the wrist of the body, and a record was made of various details about the scene of discovery, including, e.g., insects, clothing, and season. This procedure enabled systematic data collection and formed the basis for the present study. The aims of this study were to (i) examine the influence of temperature on the progression of the post-mortem changes between post-mortem examination at the scene of discovery and the autopsy and (ii) identify other variables that can lead to a higher degree of post-mortem changes during this period.

## Materials and methods

### Data basis

The Ethics Committee of the Goethe University (ethical approval number 116714) approved this study. All experiments and methods were conducted in accordance with the guidelines and regulations of the ethics committee. Informed consent was waived by an Institutional Review Board (IRB). This study included cases from the Institute of Legal Medicine in Frankfurt am Main, in which forensic pathologists conducted both the post-mortem examination at the scene of death and the autopsy. Following discovery and a first examination, the bodies were placed in white plastic body bags and transferred to a mortuary, where they were stored under refrigeration (6 °C to 7 °C) for varying periods of time. The bodies were then transferred to the Institute of Legal Medicine in Frankfurt am Main, where they were maintained under controlled refrigeration (6 °C) for variable durations until their autopsy. Overall, 135 cases were examined between May 2022 and April 2024. For each case, the following parameters were gathered:

- Date (day, month, year) of the discovery.
- Sex, age, and clothing of the deceased.
- Place of discovery (indoor, outdoor).
- Insect colonization on the body (yes, no).
- Time period from discovery until autopsy.
- Duration of cooling in the mortuary and in the cooler of the Institute of Legal Medicine.
- Presumed PMI, based on the time of last seen alive or other indications such as newspapers, letters, or statements from neighbours and relatives.
- Manner of death (natural, unnatural, unclear).

### Temperature recordings

For 94 bodies, continuous recordings of the temperature inside the body bags from the first examination until autopsy were available. The temperature was measured with one iButton (DS1922L-F5, Maxim Integrated, San Jose, CA, USA) secured to the wrists of the bodies. The temperature of each body was measured hourly and a temperature profile for the entire cooling period from the time of discovery until autopsy was established. To enable a clearer comparison of the cooling process over time, two different threshold temperatures were utilised, namely 6 °C and 10 °C. The choice of those threshold temperatures was based on the body temperatures commonly reached during storage at the mortuary (10 °C) and in the cooler at the Institute of Legal Medicine (6 °C). The cooling temperatures set in the morgue and in the Institute of Forensic Medicine in Frankfurt am Main were 6 °C.

### Documentation the post-mortem changes

For each body, a standardised set of photographs was obtained both on the day of discovery and during the autopsy to record the post-mortem changes present at each point in time. This scheme included photographs of the surrounding environment at the scene, portrait pictures of the head and neck, and comprehensive overviews of the trunk, arms, and legs from both anterior and posterior perspectives. The pictures were captured by the attending forensic pathologist and served as the basis for the documentation and quantitative scoring of decomposition, thereby reflecting the post-mortem changes. The assessment of all photographs was conducted by one author, whereas the pictures were taken by several authors.

The state of decomposition of the bodies was evaluated using two different scoring systems. The first system represents a modification of the method originally devised by Galloway et al.^24^ and further developed by Megyesi et al.^1^, in which the decomposition is categorized into four primary stages: fresh, early decomposition, advanced decomposition, and skeletisation. Within each main category, several sub-items describe specific post-mortem changes in detail, each of which is assigned a numerical value ranging from 1 to a maximum between 10 and 13. The body is divided into the three anatomical regions: head and neck, limbs, and trunk. For each region, the values corresponding to the observed post-mortem changes are recorded. The summation of these values is then used to calculate the TBS, which serves as a quantitative measurement of the overall decomposition state^1^. The second system is a modified version of the Megyesi et al.^1^ scoring method, further developed by Gelderman et al.^48^. In this method, the body is also divided into the same three anatomical regions. Every region is allocated six stadia of decomposition with corresponding scores ranging from one to six. The different stadia include multiple post-mortem changes, which are not given a separate score. Subsequently, the scores are recorded for each body region and then cumulatively calculated to derive the Total Decomposition Score (TDS)^48^. This method views decomposition as a spectrum rather than a sequential process. In this regard, the various post-mortem changes can occur simultaneously or in a slightly different order.

### Statistical analysis

The influence of various factors on the progression of post-mortem changes (measured in the increase in TDS/TBS) between the scene of death and the autopsy was examined. Complete datasets, including all recorded variables required for the Generalized Additive Models (GAMs), were available for 104 bodies. The GAM was applied to investigate the factors influencing the post-mortem changes of a body between the scene of death and at autopsy. The model was fitted using thin plate regression splines to flexibly capture non-linear relationships. Smoothing parameters were estimated via Restricted Maximum Likelihood (REML). The difference in TBS/TDS values between post-mortem examination and autopsy was used as the dependent variable in the model. Season, insect colonization, cooling time, site, PMI, initial body temperature, clothing, day of the year, and closure conditions were included as independent variables, while individual bodies (cases) were modeled as random effects to account for subject-specific variability. Separate GAMs were performed for each method of quantifying post-mortem changes, as described by Gelderman et al.^48^ and Megyesi et al.^1^. Initially, all the possible independent variables were included and then the most suitable model was identified using the Akaike Information Criterion (AIC) with a threshold of two. Variables that did not contribute to model fit (i.e., negatively impacted the AIC) were subsequently removed. The final model represented a balance between a low AIC value, model simplification, and the substantive relevance of the variables. The statistical software R Studio^54^ was used for analysing the data and creating the plots.

**Fig. 1:**
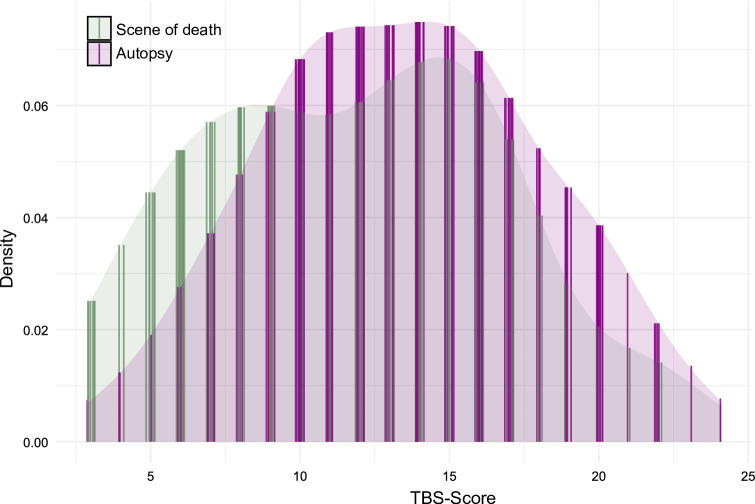
Kernel-Density-Estimation (KDE) of the post-mortem changes classified according to the Megyesi et al. system. Comparison between scene (green) and autopsy (purple).

**Fig. 2:**
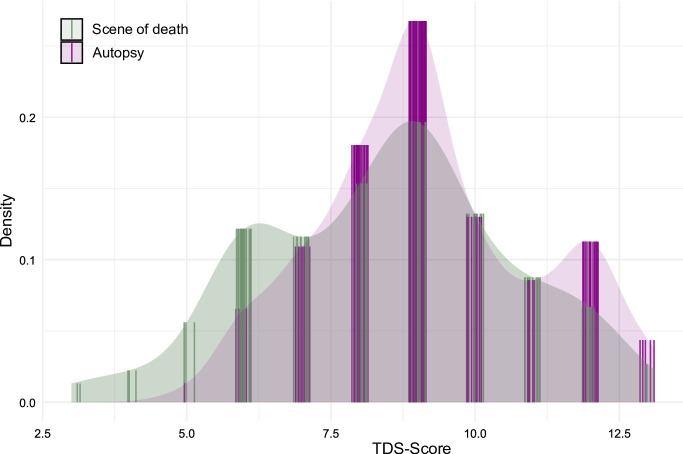
Kernel-Density-Estimation (KDE) of the post-mortem changes classified according to the Gelderman et al. system. Comparison between scene (green) and autopsy (purple).

To visualize the distribution of the non-parametric TBS and TDS scores, Kernel Density Estimation (KDE) was employed. For each combination of point of time (scene of death vs. autopsy) and scoring system (Megyesi^1^ vs. Gelderman^48^, the corresponding KDE was calculated. Estimated densities at the measurement points were determined using linear interpolation, and the values were slightly jittered to improve clarity in the figures (see Figs. 1 and 2).

## Results

### Case evaluation

A total of 135 cases were examined. Hourly temperature profiles were available for 94 bodies, in addition to their initial body temperature. The study included 102 male and 33 female bodies, ranging in age from 23 to 99. The mean age recorded was 58 years, with a median age of 59. 117 bodies were found indoors, whereas 18 were found outdoors. 37 bodies were discovered in spring (March to May), 41 in summer (June to August), 32 in autumn (September to November), and 25 in winter (December to February). 61 bodies were infested with insects. The PMI, as determined based on police reports, ranged from 0 to 113 days with an average of 14.7 days and a median of 5 days. In eight cases, the post-mortem interval could not be established due to an absence of sufficient evidence. The manner of death was categorised as natural in 60 cases, unnatural in 32 cases, and unclear in 43 cases. The average post-mortem interval for unexplained causes of death was 28.6 days. The average post-mortem interval for an explained cause of death was 9.9 days.

### Temperature profile

**Fig. 3:**
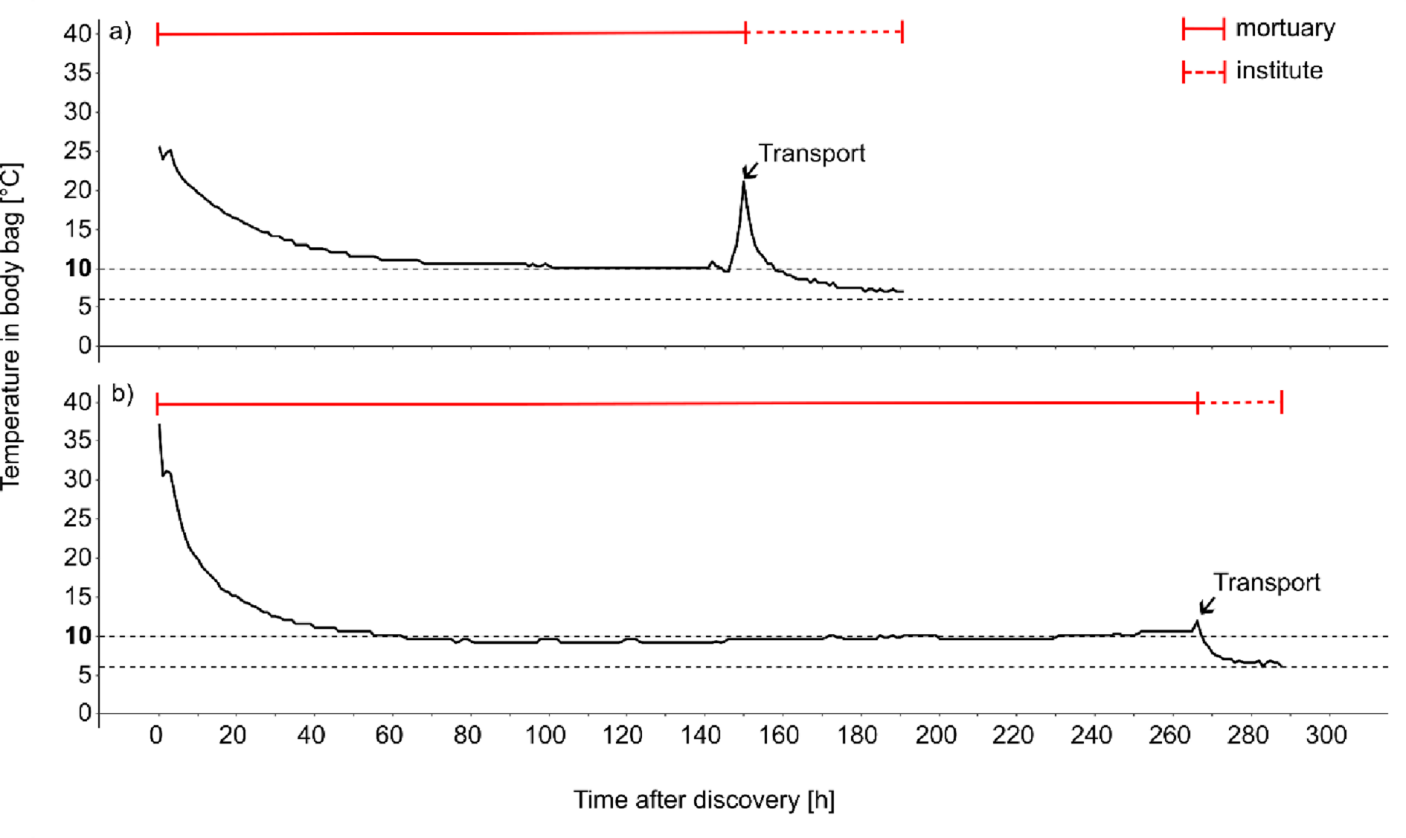
Hourly temperature inside body bags from the time of discovery at the scene of death until autopsy. Both dashed black lines show the two temperature thresholds of 6 °C and 10 °C. The red lines present the entire cooling period, separated into the time stored at the mortuary and the Institute of Legal Medicine. The arrow shows the transport from the mortuary to the Institute. (**a**) Body of an 84-year-old woman with a PMI of 32 days and a cooling time of 8 days found indoors during summer, colonised with insects. (**b**) Body of an 85-year-old man with a PMI of 3 days and a cooling time of 12 days, found outdoors during summer, colonised with insects.

The total cooling and storage period was between 1 and 15 days, with a mean duration of 6.6 days. While the cooling period at the mortuary lasted between 0 and 14 days (average of 4 days), cooling at the Institute of Legal Medicine lasted between 1 and 5 days (average of 2 days). A body spent approximately 60% of the total cooling time in the mortuary, i.e., not at the Institute of Legal Medicine. A comparative analysis of these cooling profiles shows that most exhibit a similar characteristic curve. The initial high temperatures decrease exponentially, especially within the first 40 h, gradually approaching 10 °C. This is followed by a short increase during transportation from the mortuary to the Institute of Legal Medicine in Frankfurt am Main. Thereafter, a marked decrease in temperature is observed, as it gradually approaches the 6 °C threshold (Fig. 3). The average initial body temperature was 23.4 °C, and the average minimum temperature reached was 6.8 °C. On average, the 10 °C threshold was attained after 2.6 days. However, only 88 bodies reached this threshold. The 6 °C threshold was reached on average after 5.5 days, but just for 31 cases. The remaining bodies attained minimum temperatures ranging from 10,1 °C to 16 °C.

**Fig. 4:**
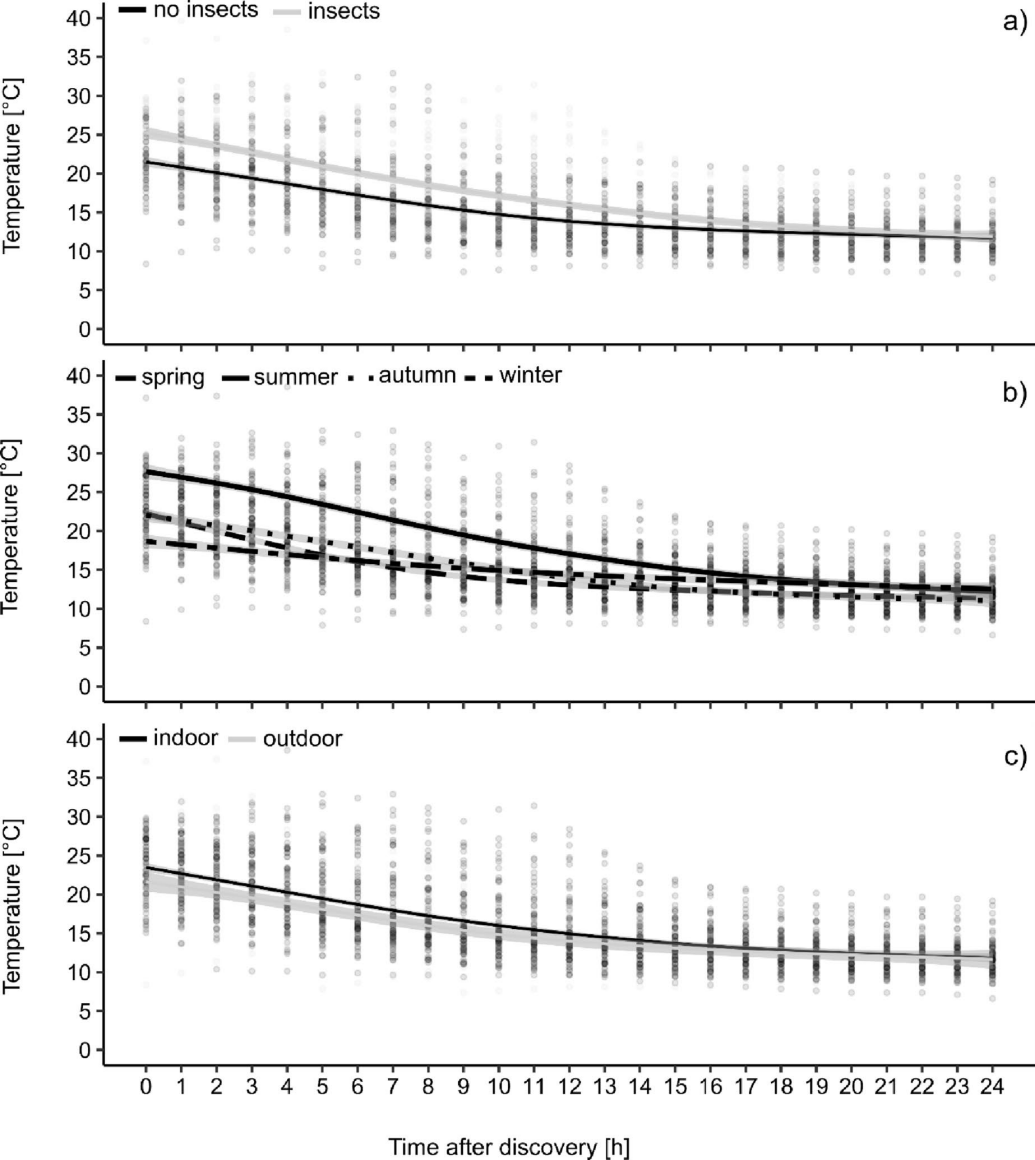
Temperatures for the first 24 h after the discovery of the body for all cases. The curves represent the average hourly temperature decline in comparison between bodies with and without insect colonization (**a**), found in different seasons (**b**), and in different locations (**c**). The gray shaded areas represent the 95% confidence intervals.

The mean initial body temperature and the maximum rate of temperature decline were significantly higher in cadavers exhibiting insect colonisation (Fig. 4). The mean body temperature of bodies colonised by insects was 11.1 °C, while the mean body temperature of non-insect-colonised bodies was 10.6 °C. The mean initial body temperature of the bodies infested with insects was 25.3 °C, whereas the mean initial body temperature of the bodies without insect infestation was 21.9 °C. After a 20-hour period, the temperature curves of infested and non-infested bodies converge (Fig. 4). A marked difference between the temperature curves is evident between the seasons (Fig. 4), showing higher initial body temperatures and more pronounced cooling rates as a characteristic of the summer months. Conversely, the curve shows a flatter trend at lower temperatures during the winter months. The spring and autumn curves fall between these two extremes. A convergence of the cooling curves of all seasons can be observed after approximately 26 h (Fig. 4). The location (indoor vs. outdoor) has a significant yet minor effect on the cooling rates of the bodies (Fig. 4), resulting in an average temperature difference of -0.8 °C for bodies discovered outdoors.

### Post-mortem changes

**Fig. 5:**
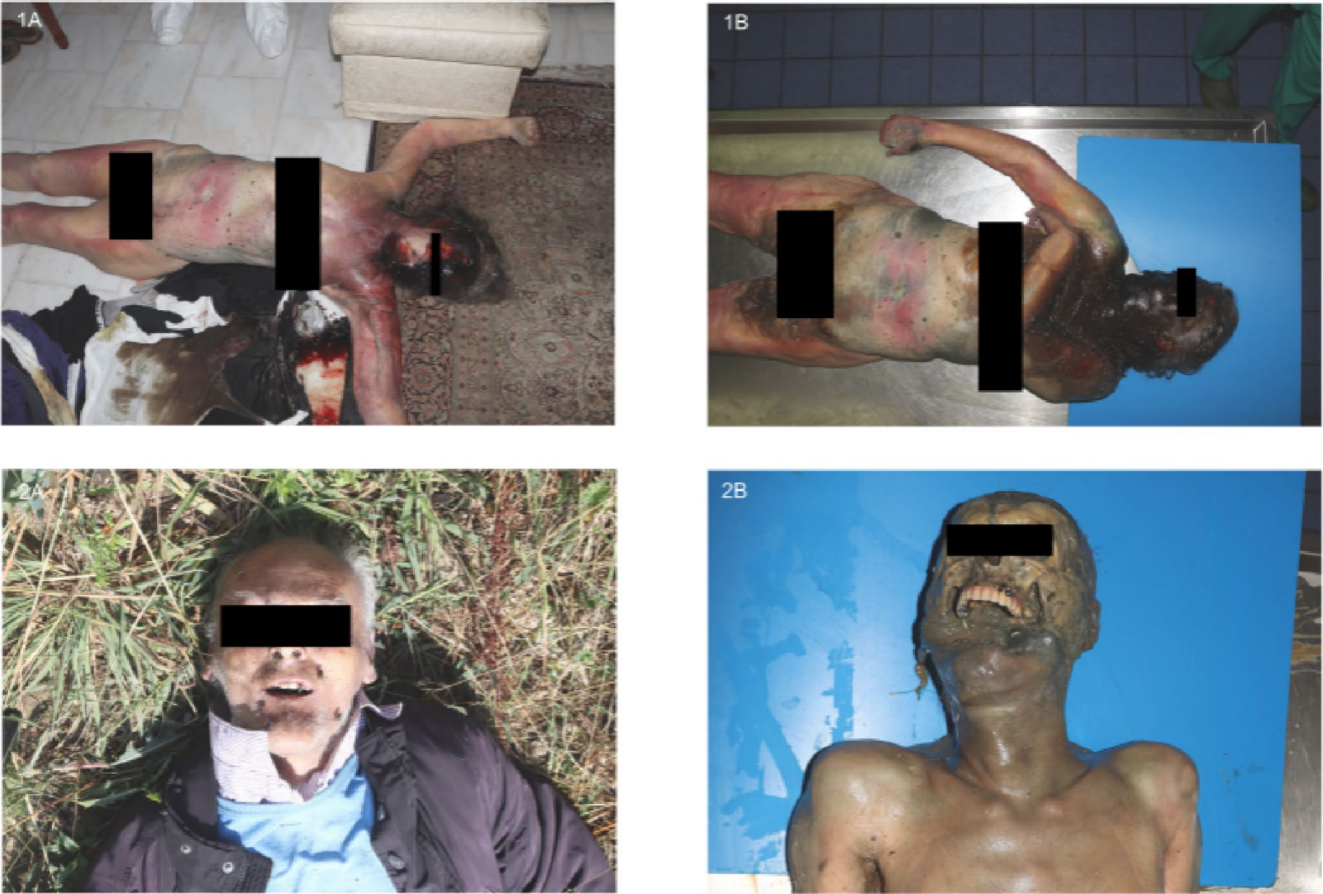
Exemplary photographic documentation from external post-mortem examination and autopsy. Body 1 corresponds to the temperature curve (a) in Fig. 1. Body 2 is a 68-year-old woman with a PMI of 7 days and a cooling time of 5 days, discovered indoors with insect colonisation during spring.

Post-mortem changes include, e.g., the discoloration of the body, bloating, or skin slippage. Extended intervals between discovery and autopsy can result in skeletisation or mummification. Using the scoring system established by Megyesi et al.^1^, 67% of cases exhibited higher TBS values at autopsy, whereas the system by Gelderman et al.^48^ indicated progression of post-mortem changes in 45% of cases. Overall, a comprehensive analysis of all post-mortem changes shows a significant increase at the time of autopsy, compared to the time of discovery, as evidenced by both evaluation systems (Figs. 1 and 2). In the comparative analysis of the individual body regions, no significant disparities in the degree of decomposition were observed between the head and neck, trunk, and limbs in either of the scoring systems. Consequently, no single body region appears markedly more susceptible to post-mortem changes than others. An example is given for the case of a man discovered in summer without signs of decomposition except for insect colonization, i.e., already eggs of blow flies in the natural orifices (Fig. 5: 2 A, 2B). At the time of autopsy, the decomposition had clearly progressed, resulting in a skeletonization of his face. Another example is the case of a woman who, when found, showed post-mortem changes in the form of brown discoloration on the left side of her face. At the time of the autopsy, the discoloration had already spread to the upper body and the entire head. The green rot on the abdomen had progressed, and the corpse was bloated (Fig. 5: 1 A, 1B).

### Key variables influencing post-mortem changes

**Fig. 6:**
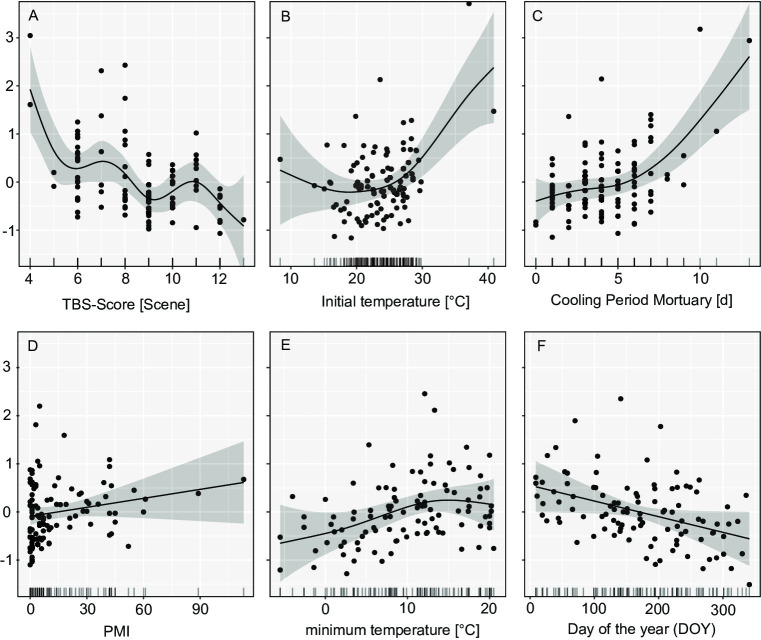
GAM Gelderman: Illustration of the effect on the progression of post-mortem changes in various variables (y-axis) modelled using Generalised Additive Models (GAMs). The black lines show the effects estimated by GAMs; the shaded areas correspond to the 95% confidence intervals. Each point represents an observation.

Using the scoring method developed by Gelderman et al.^48^, the progression of the post-mortem changes is primarily influenced by three key factors: the initial extent of post-mortem changes, elevated initial body temperatures, and longer cooling durations at the mortuary (Fig. 6; Table 1). Especially for cases with minimal post-mortem changes at the time of discovery, the model predicted a greater increase in post-mortem changes during the cooling period prior to autopsy, indicating that bodies in the fresh stage of decomposition are at higher risk for substantial post-mortem progression. Bodies that were nude at the time of discovery were significantly associated with a reduced TBS difference (discovery versus autopsy) throughout the cooling period (Table 2). A seasonal effect was also observed: the difference in post-mortem changes increased less in spring and summer than in autumn (Table 2). The length of the PMI and the minimum ambient temperature did not exert a significant influence on the post-mortem changes. The model accounts for approximately 52.7% of the adjusted variance. The explained deviance is 62.3%, which indicates a satisfactory model fit, particularly within a biological or forensic context.

**Table 1:** F-values from generalized additive models (GAMs). Comparison of the two different model approaches, Megyesi and Gelderman. This table shows which influencing variables (model parameters) contribute significantly to the respective models.

Model parameter	F-values of parameters included in the models (edf value)	
	Megyesi System	Gelderman System
TBS-value at the scene	18.891 (1.00) ***	4.657 (5.68) ***
Initial temperature [°C]	4.092 (2.74) **	5.099 (3.13) **
Cooling period in the mortuary [d]	4.824 (2.67) **	7.680 (2.79) ***
Post-Mortem-Interval (PMI)	0.679 (1.00) ^ns^	2.026 (1.00) ^ns^
Mean daily temperature [°C]	0.853 (1.00) ^ns^	-
Minimum daily temperature [°C]	-	2.290 (2.34) ^ns^
Day of the year (DOY)	-	4.010 (1.00) *

Note: p-value *** < 0.0001; ** < 0.001; *< 0.05; ns = not significant.

**Table 2:** T-values of the generalized additive models (GAMs) of the categorical parameters for the Megyesi and Gelderman systems. The reference category is fully clothed in autumn.

Model parameter^1^	Estimate with SE of parameters included in the models (t- value)	
	Megyesi System	Gelderman System
(Intercept)	2.7068 ± 0.47 (5.7)***	1.50265 ± 0.26 (5.73) ***
No insects	-1.0160 ± 0.48 (-2.08) *	-
Season		
spring	-0.8055 ± 0.46 (-1.72) **	-0.9194 ± 0.32 (-2.18)**
summer	-1.0140 ± 0.59 (-1.71) ^ns^	-0.9839 ± 0.32 (-3.00) **
winter	0.2795 ± 0.61 (0.45) ^ns^	-0.6941 ± 0.42 (-1.64)
clothing		
naked	-	-0.51524 ± 0.26 (-2.34)*
partially	-	-0.08114 ± 0.21 (-0.51)

Note: ^1^ the reference category is fully clothed in autumn; p-value *** < 0.0001; ** < 0.001; *< 0.05; ns = not significant.

**Fig. 7:**
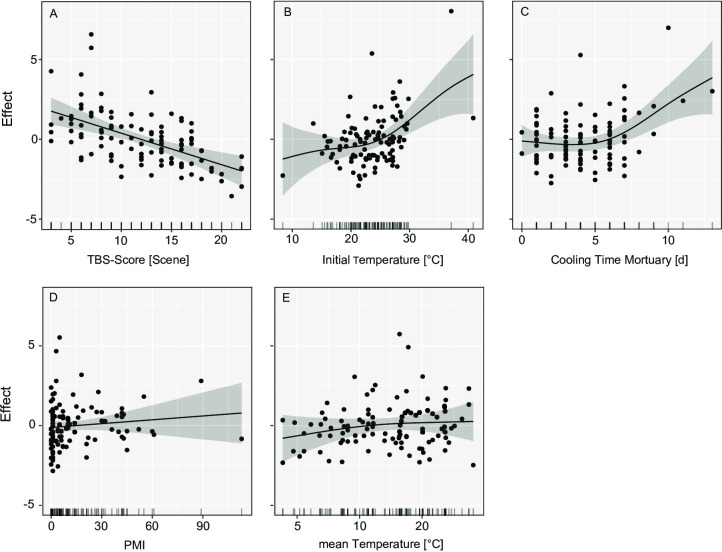
GAM Megyesi: Illustration of the effect on the progression of post-mortem changes in various variables (y-axis) modelled using Generalised Additive Models (GAMs). The black lines show the effects estimated by GAMs; the shaded areas correspond to the 95% confidence intervals. Each point represents an observation.

Using the scoring method developed by Megyesi et al.^1^, the same three variables significantly influence the progression of post-mortem changes (Fig. 7; Table 1). A nearly linear relationship was observed between the initial TBS value and the extent of post-mortem progression, again demonstrating that bodies in early decomposition stages are more susceptible to marked change during cooling. Neither PMI nor average ambient temperature reached statistical significance. However, the colonization of a body by insects was correlated with a significant positive influence on the extent of later post-mortem changes, i.e., bodies colonized with insects decomposed faster even during the cooling period. Seasonal effects remained significant, with springtime cases showing a smaller increase in decomposition compared with autumn (Table 2). A strong positive effect on the progression of post-mortem changes can be observed, particularly after a cooling period of > 5 days at the mortuary and an initial body temperature of above 25 °C. The model explained 51% of the total variance in the response variable, confirming its robustness.

## Discussion

### Interpretation of the key results

The findings of this study demonstrate that the initial body temperature and the post-mortem changes, i.e., stage of decomposition, at the time of discovery, as well as the duration of the cooling period at the mortuary, are key variables influencing the progression of post-mortem changes, independent of the system used (Gelderman et al.^48^ or Megyesi et al.^1^.

The results of the study at hand further show that bodies colonized with insects exhibit greater post-mortem changes at the time of autopsy, compared with the scene of death, than bodies without insect activity. These observations are consistent with previous studies reporting higher TBS values and faster decomposition rates in bodies infested with insects^2,29,37^. One plausible explanation is the creation of a microclimate by maggot masses, i.e., local higher temperatures above the ambient temperature, due to their metabolic activity. The study at hand found that the mean body temperature of insect-infested bodies averaged 5,1 °C above the set cooler temperature. Huntington et al.^55^ found that the temperature of bodies infested with insects averaged 8 °C above the cooler temperature. Lutz et al.^51^ had similar results with temperatures around 10 °C higher inside the body bag of insect-infested bodies than the baseline temperature of the cooler. Their study also found that after removal of the majority of larvae, body temperatures declined more rapidly^51^. Furthermore, recent research indicates that egg laying alone is sufficient, as insects can continue to develop, even when stored under refrigeration^56^. In addition, maggots feed on the soft tissue of a corpse, which also leads to progressive post-mortem changes^55^.

The observation that higher initial body temperatures result in more pronounced post-mortem changes is in agreement with prior research^1,36^. Elevated temperatures promote decomposition by enhancing enzymatic activities, bacterial growth, and insect activity, thereby accelerating the progression of post-mortem changes. Conversely, lower temperatures reduce the rate of enzymes and denature and destroy the essential enteric bacteria required for the process of putrefaction^25^. High initial temperatures can also lead to faster protein degeneration, which may negatively impact subsequent biochemical tests^11^. Furthermore, bodies with higher initial temperatures require a greater duration of time to reach the 10 °C–6 °C threshold^57^. However, the rate of cooling to a given temperature is also influenced by additional factors, including BMI, clothing, and the moisture content of the clothes^57^.

Seasonal effects were also observed: post-mortem progression was significantly lower in spring compared with autumn, a finding consistent with Giles et al.^33^. Several other studies have found faster decay and higher TBS values in summer^29,36,47^ and lower TBS values and slower decay in winter^26,33,36^, but these patterns were not confirmed in our study. One potential explanation for this discrepancy could be that none of the cited studies was conducted in Germany, but in Spain, the USA, and South Africa, where different climatic conditions may prevail.

The study at hand also found that bodies in the stage of early decomposition have a greater increase in post-mortem changes during storage and cooling periods than bodies in later decomposition stages. This finding is coherent with previous study results^10,40,53^ and indicates a non-linear progression of decomposition. The sequential framework of the Megyesi et al.^1^ system limits its ability to accurately capture the decomposition dynamics during early stages. The near-linear relationship observed in our study between the initial TBS and the subsequent TBS difference is likely explained by this configuration of the system from Megyesi et al.^1^. Other studies using the system from Megyesi et al.^1^ to derive accumulated degree days (ADD) from the TDS have similarly shown that ADD tends to be overestimated in early decomposition stages and underestimated in later stages^58^. ADD is calculated by summing daily mean temperatures above a fixed threshold at which decomposition ceases, yielding a measure of accumulated heat energy. The TDS can be used to estimate ADD, and if the temperature values for the relevant period are known, the PMI can be calculated from the ADD^1^. Consequently, overestimation of ADD in early decomposition will produce PMI estimates that are systematically too long (i.e., overestimated), whereas underestimation of ADD in advanced decomposition will yield PMIs that are too short. The non-linear course of decomposition observed in our study, recorded with the rigid system from Megyesi et al.^1^, may also be responsible for the underestimation or overestimation of ADD. Overestimation of ADD in the early stages of decomposition could be explained by the accelerated rate of decomposition during this period. In advanced stages, however, decomposition proceeds more slowly, which may lead to an underestimation of the ADD.

There was no significant difference in the increase in decomposition between the individual body regions, head and neck, limbs, and trunk, meaning that no body region is particularly vulnerable to a more rapid progression of the post-mortem changes. This finding aligns with the conclusions of earlier research^31,53^.

### Post-mortem changes and determination of the manner of death

The present study revealed that, in approximately 55% of the cases, the post-mortem changes progressed between post-mortem examination and autopsy. This often resulted in more advanced stages of decomposition, complicating the determination of both cause and manner of death. Consequently, this may contribute to the relatively high proportion of cases with undetermined cause and manner of death (31%) observed in the underlying study. The specific patient population autopsied at the Institute of Legal Medicine in Frankfurt am Main may partially account for this proportion. In cases where bodies already exhibit marked decomposition, determining the underlying cause of death can be particularly challenging. Even with the expertise of a forensic pathologist, significant decomposition may prevent a definitive conclusion due to the loss of crucial forensic evidence. In contrast, in many deaths occurring at home or in medical facilities, the cause of death seems clinically to be evident, and the body is therefore not referred for forensic autopsy. Instead, the post-mortem examination is performed by the attending physician, who certifies a natural manner of death. Such cases are not included in the case statistics of the Institute of Legal Medicine in Frankfurt am Main, resulting in a higher relative proportion of cases with unexplained causes of death within the study at hand.

### TBS-Scoring methods in forensic routine

The TBS score is utilised in numerous studies to record post-mortem changes^28,30,53^. However, this method is not the gold standard in routine forensic work or in determining the PMI. The so-called complex method, as outlined by Madea and Brinkmann^59^, is employed in current practice^60^. This method combines a variety of techniques for determining the PMI.

As with other authors, there were practical challenges when applying the Megyesi et al.^1^ classification to document the post-mortem changes. The occurrence of characteristics such as colour did not always follow a specific sequence and stages of decomposition can even be reversed, since, e.g., mummified tissues can rehydrate to a moist decomposition stage^30,43,49^. The system after Megyesi et al.^1^ assumes that mummification and skeletisation are the final stages of decomposition, yet recent studies indicate that desiccation and moist decomposition can occur simultaneously, rather than sequentially^52^. Certain decomposition phenomena, such as saponification, are not addressed in the system^31^. Furthermore, varying stages of decomposition were sometimes observed on the same body region. When this occurred, the most advanced stage was consistently recorded for scoring purposes.

The evaluation of skin colour alterations utilised by both scoring systems was most likely developed for Caucasian individuals. The application of the classification to people of colour was not consistent due to the inability to recognise certain pigmentation characteristics on darker skin types, such as pink-white appearances or grey discolouration. It is therefore essential that a system, which encompasses a wider spectrum of skin colours, be implemented in this context. Alternatively, colour descriptions could be excluded from the classification system, as proposed by Ribéreau-Gayon et al.^43^.

A further issue was the inconsistency in the quality of the photographic documentation of the post-mortem examination and autopsy. Not all bodies had fully unclothed pictures from both the anterior and posterior perspectives, including close-up views of all relevant anatomical regions. This may have resulted in certain post-mortem changes being undocumented, thereby artificially increasing the difference in TBS/TDS scores between discovery and autopsy. Such bias may explain why nude bodies in our study were significantly associated with smaller TBS differences, in contrast to previous studies reporting slower decomposition in fully clothed bodies^26^. Other authors, however, argue that clothing slows down the cooling of a body and therefore increases the speed of decomposition^39^. Furthermore, factors such as lighting conditions during photography and mechanical damage to the skin during the storage of bodies can result in variations in TBS scores between cadaveric examination and autopsy.

Megyesi et al.^1^ established their system on bodies found outdoors. In the present study, only 18 of the 135 bodies were found outdoors. Galloway et al.^26^ discovered that decomposition indoors follows a distinct and often delayed pattern. The low number of outdoor bodies in our study may be insufficient to detect differences between indoor and outdoor decomposition^2^. It became apparent that insect colonisation is correlated with a faster progression of the decomposition. However, Megyesi et al.^1^ did not include insect colonisation in the TBS method, an issue already raised by Ceciliason et al.^2^. They emphasised that when applying TBS in an indoor setting, the model requires distinct inclusion criteria and a defined population, which will be a task for future studies.

### Storage time and conditions

Our study showed a strong correlation between the duration of the cooling period, especially at the mortuary, and the increase in the post-mortem changes. Contrary to that, Giles et al.^53^ found only a weak negative correlation between the mortuary time lag (the time a body was stored at the mortuary between discovery and autopsy) and the increase of TDS. A possible explanation for this discrepancy could be found in the different cooling conditions. Due to the absence of binding regulations (see legal requirements), it cannot be excluded that the cadavers in the study at hand were refrigerated inadequately or insufficiently during this period. In contrast, Giles et al.^53^ report that the cadavers were stored in a mortuary refrigerator at 4 °C until autopsy. Other studies confirmed that the putrefaction process and associated gas formation can continue even under controlled cooling conditions at 4 °C to 5 °C^61^. This effect is particularly pronounced if the bodies are additionally colonised with insects and stored for a prolonged duration, leading to considerable tissue loss and advanced post-mortem changes^51,55^. Huntington et al.^55^ found that the temperatures in the cooler can fluctuate by up to 6 °C due to the opening and closing of doors by staff members, in addition to the on-and-off switching of cooling compressors, resulting in suboptimal cooling. In contrast, the enhanced cooling observed at the Institute of Legal Medicine in Frankfurt am Main could be attributed to the conditions present on site. The bodies are not stored in a large room, but rather in cooling cells. The cooling cells are only opened when a body is to be moved. This is an essential component in ensuring consistent temperatures, thereby mitigating the effects of temperature fluctuations caused by entering and exiting the room. The temperature of the refrigeration system at the Institute of Legal Medicine Frankfurt am Main is set to 6 °C, whereas in the mortuary it is set to 6 –7 °C. Furthermore, the cooling periods at the Institute for Legal Medicine (average of 2 days) were shorter than the periods at the mortuary (average of 4 days). The distribution of cooling time was, on average, 60% of the total time at the mortuary and 40% of the total time at the Institute of Legal Medicine.

It is imperative to consider not only the temperature during refrigeration but also the duration of storage itself. The prolonged refrigeration times (average 6.6 days) can be attributed to several factors. Firstly, the public prosecuting office determines the necessity for an autopsy, which can result in an initial delay. Furthermore, due to the limited capacity of the cold storage facilities and the high number of autopsies performed, it is not feasible to deliver all bodies directly to the Institute of Legal Medicine in Frankfurt am Main. Instead, a temporary storage arrangement is necessary at the mortuary, which must be organized by the competent investigative authorities. Thirdly, the present study evaluated routine cases from everyday forensic practice. In cases of homicide, autopsies are typically conducted with greater expediency, generally within the first 24 h following the discovery of the body.

In accordance with Giles et al.^53^, the study at hand similarly concluded that the ambient temperature at the scene exerts no influence on the increase in the difference between TDS from the scene and the autopsy. In some cases, the ambient temperature may deviate significantly from the actual temperature of the body. This variation can be attributed to numerous factors, including but not limited to insect infestation, solar radiation, the locking conditions, and the PMI^14,55^. This provides a potential explanation for the observed lack of significant influence of this factor on the progression of post-mortem changes.

### Strengths and limitations

A major strength of the present study is that body temperature was measured directly within the body bag. This offers enhanced accuracy compared to methods relying on the ambient temperature or the mean temperature of the nearest meteorological station^62^ since the decomposition process itself generates heat, which additionally increases the body temperature^40,41^.

Using the system from Megyesi et al.^1^, which provides a clear and reproducible framework with a high interobserver reliability for recording and quantifying human decomposition, ensures comparability with other studies^49^. Furthermore, by analysing a substantial number of cases within this standardised framework, the study not only strengthens the robustness of its findings but also underscores their generalizability and applicability to a broad range of forensic contexts. A notable limitation of the study is the reliance on TBS scores recorded by a single author, which may introduce bias. The evaluation process would have been strengthened by the incorporation of an additional assessment by a second person.

The underlying study´s limitations include the evaluation of post-mortem changes in aquatic and hanging bodies with a small sample size (*n* = 4). The external manifestation of post-mortem changes in these cases differs from the terrestrial decomposition process^31^, potentially distorting the results. Hanging promotes dehydration, due to full exposure to environmental factors such as wind and sunlight^66^ and restricts insect access as non-flying insects cannot reach the body and fallen maggots cannot return. Consequently, desiccation and limited accessibility reduce insect infestation^66^. To accurately assess post-mortem changes in these special settings, a score specifically designed for this purpose should be used^63–65^. To facilitate a more effective comparison, all bodies in the present study were quantified using the same scores. Moreover, larger sample sizes are required to draw reliable conclusions about the progression of the post-mortem changes between scene and autopsy in these special settings. Further studies should investigate the progression of decomposition in such cases more closely.

Finally, the quality of PMI estimates is contingent on the evidence available for consideration^67^. For instance, a daily newspaper found at the scene may provide a less reliable time reference than the analysis of a cell phone. In several cases, the PMI interval was determined based on “last seen alive” statements of witnesses, resulting in an overestimation of the time elapsed since death, as the individual may not have perished immediately after their last sighting. This could be a possible explanation for the absence of a correlation between PMI and post-mortem changes in the study at hand.

### Legal requirements

In only three of the 16 federal states in Germany (Table 3), the Cemetery and Burial Act (Friedhofs- und Bestattungsgesetz) specifies requirements for the cooling temperature of mortuaries’ cold storage facilities. In all the other federal states, the Cemetery and Burial Act only stipulates that bodies have to be kept in suitable cooling rooms, without specification of a temperature limit. DIN EN 15,017^68^, which is intended to standardise the requirements for funeral services throughout Europe, recommends that bodies should be stored at temperatures between 0 °C and 5 °C. However, this is only a recommendation and is not mandatory. In most federal states in Germany, the respective Cemetery and Burial Act allows a body to remain unrefrigerated for up to 36 h. Cockle and Bell^25^ state that bodies exposed to lower ambient temperatures need a larger accumulation of temperature and therefore more time to reach each stage of decomposition compared to bodies exposed to higher ambient temperatures. This finding, along with the results of our study, further emphasises the importance of mandatory cooling guidelines for bodies as a method to slow down decomposition and preserve forensic evidence.

**Table 3:** Overview of the legal regulations governing temperature and time for transferring a body to a Mortuary in the cemetery and burial laws of the federal States of Germany.

Federal State	Temperature	Timeframe for transfer to the mortuary
Baden-Württemberg	-	36 h
Bavaria	-	-
Berlin	< 10 °C	-
Brandenburg	-	24 h
Bremen	-	36 h
Hamburg	-	36 h
Hesse	-	36 h
Mecklenburg-Western Pomerania	DIN 1507	36 h
Lower Saxony	-	36 h
North Rhine-Westphalia	-	36 h
Rhineland-Palatinate	-	36 h
Saarland	-	36 h
Saxony	≤ 8 °C	24 h
Saxony-Anhalt	-	36 h
Schleswig-Holstein	-	36 h
Thuringia	-	48 h

## Conclusion

Our study found that pre-existing post-mortem changes, the duration of refrigeration (especially at the mortuary), and the initial temperature of the body significantly influence the progression of post-mortem changes between discovery and autopsy. Additionally, in the cases examined in this study, an average of 5.5 days of refrigeration is required to reach a temperature of 6 °C. This finding has important implications for PMI estimations that incorporate the cooling period. When calculating the PMI using the ADD method, it cannot be assumed that the body temperature is already 4 °C when the body is placed in cold storage and that this consequently halts the onset of putrefaction. It was found that the initial body temperature was higher for bodies covered with insects and those discovered during the summer months.

To improve preservation of forensic evidence, it is crucial to consider the aforementioned factors and prioritize bodies that are at the highest risk for rapid post-mortem changes in the planning of autopsies. More specifically, bodies with an initial body temperature of > 25 °C that have been stored in a mortuary refrigerator for more than five days are particularly at risk of having significantly progressive post-mortem changes. Therefore, the establishment of standardized regulations concerning cooling temperature and duration is crucial to ensure the quality of the preservation of forensic evidence. The storage duration of bodies should be limited to five days, with a maximum body temperature of 6 °C. Further investigations are required to ascertain the conditions that must be met to ensure such body temperatures are achieved.

The following recommendations are proposed for the forensic routine:

- Uniform Germany-wide regulation for cooling temperatures at the mortuary around 4 °C.
- Prioritization of bodies with an initial body temperature ≥ 25 °C.
- Prioritization of bodies with insect colonization.
- Avoidance of storage times longer than 5 days at the mortuary.
- Avoidance of long cooling periods, especially for bodies in the early stages of decomposition.

## Data Availability

The datasets generated during and/or analyzed during the current study are not publicly available due to possible inferences about sensitive personal data but are available from the corresponding author on reasonable request.
